# From Invasive Bramble to Functional Browse: A Translational Review of *Rubus ulmifolius* Schott for Sheep Nutrition

**DOI:** 10.3390/plants15172656

**Published:** 2026-08-30

**Authors:** David Cancino-Baier, John Quiñones-Diaz, Rommy Diaz, Erwin Muñoz-Acuña, Nestor Sepúlveda Becker, Erwin A. Paz, Alex Muñoz-Salvo

**Affiliations:** 1 Facultad de Ciencias Agropecuarias y Medioambiente, Departamento de Biotecnología y Ciencias Veterinarias, Universidad de La Frontera, Temuco 4811230, Chile; 2 Laboratorio de Reproducción, Centro de Excelencia en Biotecnología de la Reproducción (CEBIOR), Universidad de La Frontera, Temuco 4811230, Chile; 3 The Marshall Centre for Infectious Diseases Research and Training, University of Western Australia, Perth 6009, Australia; 4 Programa de Doctorado en Ciencias Agroalimentarias y Medioambiente, Universidad de La Frontera, Temuco 4780000, Chile

**Keywords:** *Rubus ulmifolius*, sheep nutrition, invasive biomass, candidate functional browse, condensed tannins, feed safety, no-spread biosecurity, circular bioeconomy

## Abstract

*Rubus ulmifolius* Schott is an invasive bramble whose recurrently removed biomass may contain nutritionally useful leaves and young shoots. This narrative and translational review evaluates whether that biomass can be investigated as a candidate sheep supplement without encouraging its spread. Species-specific evidence shows marked seasonal and fraction-dependent variation in crude protein, fiber, lignin and tannin activity, whereas controlled sheep-feeding data remain scarce. Goat browsing observations and general studies of tannin-rich feeds support only indirect hypotheses regarding intake, rumen nitrogen metabolism, methane, microbiome composition, oxidative status, product quality and parasite resilience. Key constraints include mature-cane lignification, thorns and selective refusal, uncertain dose–response, processing effects, contamination and propagule biosecurity. We therefore define functional browse as an unvalidated, outcome-dependent designation and propose a staged evidence pathway: local biomass mapping and no-spread harvest; fraction-specific chemical and phytochemical characterization; in vitro digestibility, polyethylene-glycol and methane screening; short-term sheep acceptability and safety; controlled dose–response trials; targeted mechanistic studies; and farm-scale techno-economic and ecological validation. Current evidence supports *R. ulmifolius* as a candidate for structured evaluation, not as a proven practical feed. Any future use should be local, traceable, fruit-free or seed-inactivated, and contingent on maintained intake, digestibility, welfare and performance.

## 1. Introduction: An Invasive Bramble as a Candidate Sheep-Feed Resource

Invasive alien plants are major ecological and economic stressors, causing biodiversity loss, habitat transformation, reduced agricultural accessibility and recurrent control costs [1,2]. *Rubus ulmifolius* Schott (elmleaf or wild blackberry) is a particularly relevant case because it forms persistent thorny thickets, is consumed selectively by browsing ruminants and contains tannins and other phenolic compounds that could influence feed value.

Management of *R. ulmifolius* commonly relies on repeated cutting, herbicides, targeted grazing or biological control [3,4,5]. These interventions confirm the difficulty of controlling regrowth and repeatedly generate biomass that is usually discarded. Valorization could offset part of that burden only when it complements existing control, uses biomass from already invaded stands and follows a strict no-spread protocol; it must never create an incentive to cultivate or disseminate the species.

The question is relevant to extensive and semi-extensive sheep systems exposed to seasonal forage gaps and declining forage quality [6,7,8]. Shrub leaves and young shoots may buffer scarcity, but secondary metabolites are dose-dependent and can be beneficial, neutral or detrimental [9,10,11]. For *R. ulmifolius*, the season, plant fraction, lignification, tannin activity, thorns and processing are therefore as important as crude protein, and goat browsing observations cannot be used to predict sheep intake or performance directly.

**Figure 1:**
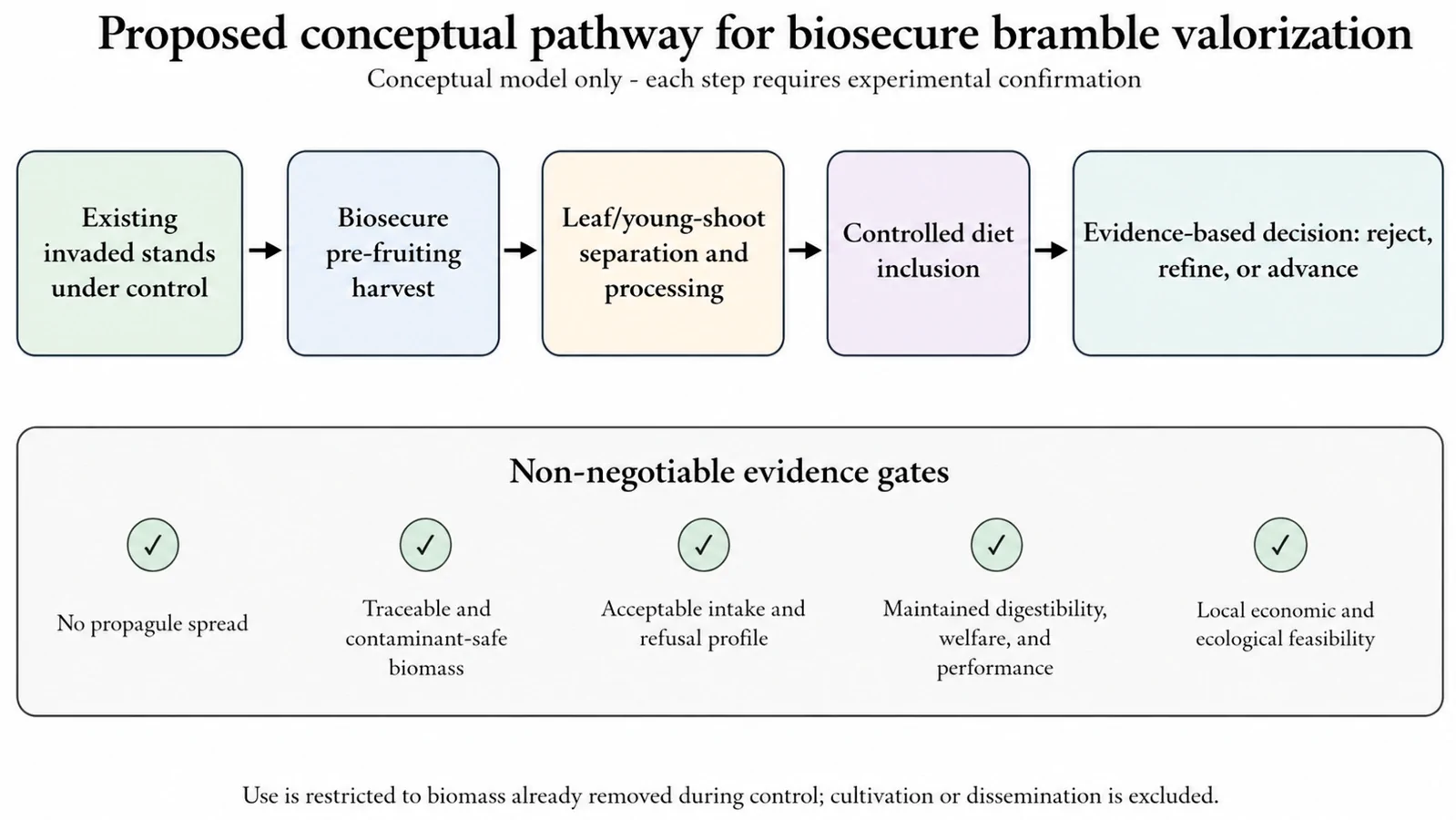
Proposed conceptual pathway for converting biomass removed from invasive *R. ulmifolius* stands into a monitored candidate sheep supplement. The pathway is not validated and requires experimental confirmation at each evidence gate. It excludes cultivation and emphasizes pre-fruiting harvest, local processing, no-spread biosecurity, controlled diet inclusion, animal safety and farm-level feasibility.

This review asks under what ecological, nutritional and operational conditions biomass removed from invasive *R. ulmifolius* stands can be evaluated safely and practically as a sheep supplement. Its objectives are to critically synthesize direct and indirect evidence, define nutritional and biosecurity boundaries, and present a staged validation framework. It does not recommend current farm feeding or cultivation; *R. ulmifolius* is treated throughout as a candidate whose functional value must be demonstrated in controlled sheep trials (Figure 1).

## 2. Review Scope, Literature Search and Evidence Hierarchy

This narrative and translational review is organized around a single question: under what biological, nutritional and operational conditions can biomass removed from invasive *R. ulmifolius* stands be evaluated as a safe and practical sheep supplement? The literature outside this question was retained only when it clarified plant identity and invasion risk, nutrient or phytochemical composition, ruminant mechanisms, processing, feed safety, biosecurity or farm feasibility.

Targeted searches were conducted in Scopus, Web of Science Core Collection, PubMed and Google Scholar from database inception to 20 July 2026. Search strings combined “*Rubus ulmifolius*” or “elmleaf blackberry” with sheep, lamb, ewe, goat, ruminant, browse, forage, feed, nutrition, digestibility, tannin, phenolic, rumen, microbiome, methane, Haemonchus, anthelmintic, invasive, control, processing, biosecurity and circular bioeconomy. Reference lists of relevant papers were screened to identify older or regional reports.

Peer-reviewed primary studies and reviews were included when they addressed *R. ulmifolius* ecology or management, species-specific chemical composition, ruminant browse use, tannin- or phenolic-mediated mechanisms, processing, safety or implementation. A historical Chilean analytical report was retained as species-specific composition evidence [12]. Direct controlled sheep studies were assigned the greatest weight. Goat studies, general ruminant studies and in vitro assays were treated as indirect evidence, while fruit-only and pharmacological extract studies were used only for phytochemical or mechanistic context. Studies with uncertain taxonomic identity or no relevant ecological, nutritional or animal endpoint were excluded.

Because the review is narrative rather than systematic, no pooled effect estimate was attempted. Evidence was separated by outcome domain in Table 1 and classified according to whether it supported a species-specific conclusion, an indirect mechanistic inference or a hypothesis requiring controlled sheep validation. Goat browsing data were not used to define sheep inclusion levels, and extract bioactivity was not interpreted as evidence of rumen or animal-health efficacy.

The resulting framework integrates three necessary conditions: use must not increase invasion risk; the harvested product must have an acceptable and reproducible nutritional and safety profile; and any proposed functional benefit must be measurable at the rumen or animal level without loss of intake, digestibility, welfare or performance. Until these conditions are demonstrated, *R. ulmifolius* should be described as a candidate rather than a validated functional feed.

## 3. Botanical Identity, Invasiveness and Management Relevance

### 3.1. Botanical Traits That Matter for Both Invasion and Feed Use

*R. ulmifolius* belongs to Rosaceae and forms perennial, thorny, arching canes with compound leaves, white-to-pink flowers and aggregate fruits. Its ability to reproduce by seeds and vegetative propagation allows it to persist in disturbed areas, field margins, riparian zones, forest gaps and abandoned land. For feed valorization, the relevant plant fractions are not the mature woody canes but leaves, petioles and young shoots, because these fractions have lower lignification, higher crude protein and a more favorable ratio between nutrients and structural fiber. The same architecture that makes the plant difficult to access also creates handling challenges for cut-and-carry feeding.

From a botanical-resource perspective, bramble biomass is heterogeneous. A sample cut from a dense thicket may contain young leaves, old leaves, petioles, immature stems, lignified stems, flowers, fruits and dead material. Nutritional values therefore depend strongly on the harvested fraction. This matters because the published values for the whole plant, browsed portions or leaves cannot be treated as interchangeable. If future studies analyze only young leaves, the reported crude protein and digestibility will probably be higher than values obtained from mechanically shredded whole canes. Conversely, if stems dominate the harvested biomass, acid detergent lignin will increase and digestibility will decline.

Thorns are a genuine feeding limitation rather than only a handling nuisance. They may reduce voluntary intake, increase sorting and refusals, irritate oral tissues and create a difference between the composition offered and the composition actually consumed. Controlled trials should therefore quantify offered feed and refusals and, where possible, separate leaf-, stem- and thorn-rich fractions before calculating *R. ulmifolius* intake.

### 3.2. Invasion Impacts and the Limitations of Conventional Control

*R. ulmifolius* forms dense thickets that can suppress native vegetation, limit forest regeneration, modify animal movement and generate repeated control costs. In Chile, evidence from Robinson Crusoe Island shows that invasive plant thresholds are closely linked to the regeneration of endemic forest species, and *R. ulmifolius* has been part of the invasive plant complex affecting conservation outcomes [4]. The pathogenicity of Phragmidium violaceum against *R. ulmifolius* has been evaluated in Chile, illustrating the need for alternatives to repeated mechanical or chemical control [3].

Conventional control options include cutting, burning, herbicide application, biological control and targeted grazing. Each option has limitations. Mechanical cutting may temporarily reduce above-ground biomass but often stimulates regrowth unless repeated. Herbicides such as glyphosate, metsulfuron, triclopyr or triclopyr–picloram mixtures may reduce cover, but they can be incompatible with organic production, sensitive habitats or public perception [5,15]. Biological control can reduce vigor but is usually slow, context-dependent and not designed to generate a usable biomass product. Targeted grazing can reduce shrub cover but requires animal management, fencing, timing and monitoring to avoid overgrazing or inadequate control.

The economic rationale for valorization emerges because control generates biomass repeatedly. In most management programs, cut biomass is treated as waste, mulch or an inconvenience. If a safe fraction of that biomass could be transformed into a feed ingredient, part of the control cost could be offset by feed value. However, valorization should never be confused with cultivation. The goal is not to promote *R. ulmifolius* planting or spread, but to create a biosecure use for biomass already removed as part of control programs.

A strict no-spread principle is therefore non-negotiable. Feed-chain protocols should prioritize harvest before fruit maturation, prevent seed dissemination, clean machinery, avoid moving viable canes into uninvaded areas, process biomass locally where feasible and manage residues so that neither seeds nor vegetative fragments establish.

### 3.3. Valorization Is Not Eradication: Defining the Realistic Management Claim

The strongest and most defensible claim is that feed valorization could complement invasive shrub management, not replace it. *R. ulmifolius* is difficult to eradicate because of its regrowth capacity and vegetative propagation. A sheep-feed strategy would not eliminate the species by itself. Instead, periodic harvesting could reduce standing biomass, improve access, lower fuel or herbicide dependence in selected contexts, and generate a local feed supplement. This is a biomass-use strategy embedded within integrated management.

The realistic hypothesis is that repeated removal of young *R. ulmifolius* biomass, integrated with established control methods, may reduce management waste while generating material for a standardized sheep supplement. This does not imply eradication and should be tested through paired field plots that measure regrowth, biomass yield, phenology, nutrient composition, propagule risk and animal response.

## 4. Sheep-Feeding Context: Forage Scarcity, Shrub Resources and the Browse Niche

### 4.1. Why Sheep Systems Need Locally Available Feed Buffers

Sheep production in pasture-based systems is vulnerable to seasonal feed gaps, drought, heat stress and fluctuations in forage quality. Climate change can affect livestock production through altered pasture growth, lower forage nutritive value, water limitation and increased heat load [6,8]. Forage quality can decline with rising temperature, with implications for digestibility and enteric emissions [7]. These pressures increase interest in locally available feed resources that can complement conventional pasture, hay or concentrates.

Non-conventional feed resources are most useful when they address a defined constraint. For sheep, the constraint may be a lack of green biomass during dry periods, high feed costs, limited protein supply, parasite pressure, or the need to reduce reliance on imported ingredients. Shrub biomass can contribute to these goals, but its value is context-dependent. Shrubs often contain higher secondary-metabolite concentrations than herbaceous forages, and these metabolites can have both nutritional and antinutritional effects [10,11,16].

*R. ulmifolius* is relevant because it may remain available when herbaceous pasture declines, especially in margins or neglected areas. However, availability alone is not enough. A feed resource must be accessible, acceptable to animals, safe, nutritionally useful and compatible with farm logistics. The review therefore treats *R. ulmifolius* as a potential buffer ingredient rather than a basal feed. This conservative positioning is essential for scientific credibility.

### 4.2. Browse Use: Lessons from Goats, Sheep and Silvopastoral Systems

Most species-specific browsing evidence comes from goats in Mediterranean systems, where Rubus spp. may be selected according to the season, availability and plant quality [17,18,19]. These observations show that bramble can be encountered and consumed by browsing ruminants, but goats differ from sheep in diet selection, woody-plant use and tolerance of tannin-rich feeds. Consequently, goat data cannot define sheep palatability, safe inclusion, digestibility or production responses.

Direct sheep evidence is much more limited. Understorey foraging in mixed Atlantic woodland supports only the broader principle that sheep can use some woody vegetation under appropriate conditions [20]. For *R. ulmifolius*, the presentation form, plant fraction, basal diet and measured refusals are likely to be more important than field availability alone.

The browse niche for sheep is therefore strategic. *R. ulmifolius* is unlikely to be ideal as a sole roughage, especially if harvested as a mature whole plant. It may be more appropriate as a moderate supplement mixed with hay, pasture, silage or concentrates. This mixed-feeding approach can dilute tannins and lignin while preserving potential benefits from protein, minerals and phenolics. The broader shrub-use literature supports this interpretation: shrub intake can be increased by managing animal experience, plant diversity, supplementation and the availability of alternative feeds [9,21].

### 4.3. Candidate Functional Browse: Definition and Minimum Evidence

In this review, functional browse means a browse ingredient intended to produce a measurable benefit beyond basal nutrient supply. For *R. ulmifolius*, this is a provisional, hypothesis-driven category rather than a validated designation. The presence of tannins, phenolics or antioxidant activity alone is insufficient to establish functional value.

Minimum evidence would require a chemically characterized and biosecure product, at least one acceptable inclusion level in sheep, maintained or improved intake, digestibility, welfare and performance, and a reproducible outcome such as improved nitrogen use, parasite resilience, rumen fermentation, methane intensity, oxidative status or product quality relative to an appropriate control. Until these criteria are met, the term candidate functional browse is used.

## 5. Nutritional Composition and Forage Value of *R. ulmifolius*

### 5.1. Chemical Composition and Seasonal Variation

Species-specific nutritional evidence is limited but includes an early Chilean analysis and a modern seasonal dataset. The 1951 Chilean report analyzed 40 bramble samples and compared proximate and mineral composition with alfalfa; however, its historical methods, reporting basis and absence of controlled animal outcomes limit modern inference [12]. The most directly relevant contemporary dataset is the seasonal study from northeastern Algeria, which measured chemical composition, secondary metabolites, digestibility and polyethylene-glycol responses in *R. ulmifolius* and three other browse lianas [13]. In that study, crude protein ranged from approximately 14.8% to 18.9% of dry matter, while fiber and acid detergent lignin increased markedly in summer.

The seasonal pattern indicates that a single fixed feed value would be misleading. Spring material combined higher crude protein with lower lignin, whereas summer material had higher NDF, ADF and ADL [13]. This supports preferential evaluation of early regrowth or spring leaf-rich biomass, but the result comes from one region and cannot be generalized without multi-site validation.

Seasonal differences may reflect both phenological maturation and environmental conditions (Table 2). Temperature and water deficit can accelerate structural maturation, while light exposure, soil fertility and moisture, disturbance or cutting history, population or genotype, and leaf position can alter nutrient and phenolic accumulation. Substantial variation among Rubus organs, species and collection sites has been reported [22,23], but these drivers have not been separated for *R. ulmifolius* browse. Future datasets should therefore record recent temperature and rainfall, soil characteristics, canopy exposure, disturbance history, phenological stage and leaf:stem ratio rather than attributing all variability to the season alone.

Crude protein alone is insufficient to establish feed value because tannin-mediated protein binding and lignin can reduce ruminal availability, cell-wall digestion and voluntary intake. Interpretation should give equal weight to NDF, ADF, ADL, in vitro and in vivo digestibility, tannin activity, protein-precipitating capacity, the refusal rate and the composition actually consumed [24,25].

### 5.2. Digestibility, Metabolizable Energy and the Role of Tannins

Digestibility is a central bottleneck. Laadjal et al. reported relatively favorable digestibility for *R. ulmifolius* and *Clematis cirrhosa* among the evaluated lianas, but the polyethylene-glycol response showed that tannins altered fermentation [13]. Because polyethylene glycol binds tannins, a positive response identifies biologically active tannins but does not by itself distinguish a useful protein-protection effect from a constraint on digestion.

Older Mediterranean browse studies likewise show that chemical composition, tannin concentration and polyethylene-glycol responses depend on the plant source and animal context [26,27]. These goat-based data help identify tannin-related constraints, but they do not establish sheep inclusion levels or performance.

The practical implication is that *R. ulmifolius* should not be evaluated only through conventional proximate analysis. A minimum nutritional panel should include total phenolics, total tannins, condensed tannins, hydrolyzable tannins where possible, protein-precipitating capacity, in vitro dry matter digestibility, in vitro organic matter digestibility, gas production kinetics, methane production and response to PEG. Without these measurements, feeding recommendations will remain speculative.

### 5.3. Plant Fraction, Harvest Timing and Processing Form

For feed development, the key question is not simply whether *R. ulmifolius* is nutritious, but which fraction should be harvested. Browsing animals naturally select leaves and young shoots; mechanical harvesting may collect much more lignified stem. This mismatch can explain why field palatability does not necessarily translate into high intake in chopped biomass. Future studies should separate at least three fractions: leaves, young green shoots and mature woody stems. Each should be analyzed independently before mixed formulations are tested.

Harvest timing should prioritize early vegetative or spring regrowth stages when crude protein is higher and lignin lower. Repeated cutting could potentially generate regrowth with a more favorable leaf-to-stem ratio, but this must be studied. A provocative but testable hypothesis is that invasive-management cutting, if timed correctly, could generate nutritionally superior regrowth biomass compared with unmanaged mature thickets. This would transform a regrowth problem into a feed-quality opportunity, provided that repeated harvest does not spread the plant.

Processing may improve handling but is not uniformly beneficial. Chopping can reduce the physical barrier of long thorny canes without eliminating sorting; grinding improves mixing but can create dust and reduce physically effective fiber; drying and pelleting improve stability and dosing but may alter phenolic extractability or tannin activity and add cost; and ensiling may soften biomass but requires validation of pH, fermentation acids, molds, mycotoxins and aerobic stability. Fresh, dried, pelleted and ensiled products should therefore be evaluated as distinct ingredients rather than assumed to be nutritionally equivalent.

### 5.4. Comparison with Other Browse and Conventional Feeds

*R. ulmifolius* should be compared with both conventional forages and other browse species. Against hay or pasture, its advantage may be local availability, its protein content during certain seasons and phenolic functionality. Against leguminous shrubs or tannin-containing forages, its advantage may be that it is already present as an invasive biomass. Its disadvantage is heterogeneity, thorns, ecological risk and uncertain sheep performance. These trade-offs should be explicitly presented rather than hidden.

The most realistic niche is partial supplementation during forage scarcity or low-to-moderate inclusion in mixed diets, not replacement of high-quality pasture, hay or balanced concentrates. Regional biomass availability, the harvest season, leaf:stem ratio, processing capacity, sheep class, basal diet and management objective will determine whether the material has any practical advantage [9].

### 5.5. From Whole Biomass to Leaf-Enriched Material: Why Fractionation Matters

A major weakness in many discussions of unconventional forage is the tendency to treat the whole plant as a single ingredient. For *R. ulmifolius*, this is scientifically inadequate. Leaves, petioles, young shoots, reproductive structures and mature canes differ in protein concentration, fiber composition, lignification, physical structure and likely phenolic profile. A sheep does not experience “*Rubus* biomass” in the abstract; it experiences a specific harvested fraction with a given particle size, thorn burden, tannin activity and digestibility. Therefore, the feed value of the species should be defined by the fraction and process, not by botanical name alone.

Leaf-enriched material is likely to be the most promising fraction because it should contain more cellular nutrients and less structural lignin than mature stems. Young shoots may also provide useful nutrients but require special attention because thorns, epidermal toughness and moisture content can affect intake and processing. Mature canes, in contrast, are more defensible as a biomass-management residue than as a high-value feed fraction. They may still contribute physically effective fiber after chopping, but their lignin content and handling difficulty are expected to limit nutritional value. This distinction should guide future sampling strategies and should also be made explicit in future research to avoid the impression that all *R. ulmifolius* biomass is equally useful.

A fraction-specific research question is therefore more informative than asking whether the species is simply a good forage: which fraction, harvested at which phenological stage, processed in which form and included at which dose provides the best balance among intake, digestibility, nitrogen use and animal health?

The analytical standard for such work should include dry matter, crude protein, ash, ether extract, NDF, ADF, ADL, soluble protein, neutral detergent insoluble protein, acid detergent insoluble protein, condensed tannins, hydrolyzable tannins, total phenolics, antioxidant capacity and in vitro digestibility. Van Soest fiber fractions remain essential for comparing forage value, while nitrogen fractionation is important because tannins may alter the ruminal availability of protein [24,28]. For functional claims, however, these classical measurements are not sufficient; they must be linked to rumen fermentation, microbial ecology and animal-level responses.

The processing form also needs to be defined (Table 3). Fresh chopped material may be useful in cut-and-carry systems but difficult to standardize. Hay or dried leaf meal would reduce moisture and facilitate formulation, but drying may alter phenolic extractability. Pelleting could improve handling and reduce selectivity, although heat and pressure may modify bioactive compounds. Ensiling may be attractive for seasonal conservation, but fermentation quality, pH, effluent losses, mold risk and palatability must be evaluated before recommending it. Therefore, future work should compare processing forms rather than assuming that field-harvested biomass can be directly incorporated into practical diets.

### 5.6. Benchmarking Against Conventional and Alternative Feeds

*R. ulmifolius* should be benchmarked against low-cost roughages and browse resources used during pasture scarcity rather than against grain or high-quality legume hay. Young leaves may provide more protein and phenolics than straw or mature grass hay, whereas high-quality pasture and alfalfa have clearer advantages in consistency, digestibility, handling and established animal responses.

The comparison is also functional rather than purely nutritional. Conventional feeds are usually formulated to meet energy and protein requirements; functional browse is included to diversify plant secondary metabolites, alter nitrogen partitioning, potentially reduce parasite pressure, or modulate rumen fermentation. This does not make *R. ulmifolius* more valuable than conventional feeds in a simple feed table. It means its value may emerge at moderate inclusion levels within mixed diets, especially where the biomass is locally abundant and otherwise treated as a management cost.

From a research perspective, the correct comparator depends on the objective. If the objective is nutritional replacement, the control should be a basal diet with equivalent crude protein and fiber. If the objective is functional supplementation, the control should be a diet matched for energy and protein but without the *Rubus*-derived phenolic fraction. If the objective is circularity, the comparator should include the current management pathway, such as mechanical removal or herbicide-based control, and the feed value of the harvested biomass. These alternative comparators lead to different conclusions and should not be mixed indiscriminately.

## 6. Phytochemical Architecture and Functional Potential

### 6.1. Major Bioactive Compounds and Their Evidentiary Relevance

Leaves and young shoots are the relevant feed fractions and contain tannins, ellagic acid derivatives, flavonols and phenolic acids, with profiles that vary by organ, population and extraction method [22,23,29,30,31] (Table 4). Fruit anthocyanins and other fruit bioactivities are retained only as background species chemistry because fruit is not the preferred browse fraction and should generally be excluded from feed biomass to reduce propagule risk.

Antioxidant, antimicrobial and antibiofilm activities reported for *R. ulmifolius* extracts demonstrate chemical bioactivity but cannot be transferred directly to the rumen or to sheep health [29,30]. The rumen is an anaerobic, buffered and functionally integrated microbial ecosystem, so the relevant question is whether ingested, processed biomass produces selective and measurable effects without impairing fiber digestion.

Accordingly, the phytochemical evidence supports mechanistic hypotheses concerning protein binding, ammonia formation, microbial ecology, biohydrogenation, oxidative status and parasite biology. It does not establish a medicinal or functional feeding effect in sheep.

### 6.2. Antioxidant and Antimicrobial Evidence: Useful but Not Sufficient

Phytochemical studies support strong antioxidant activity in aerial parts and wild Rubus leaves [23,31]. Fruit studies confirm additional species bioactivity [35] but have limited relevance to browse biomass. None of these findings demonstrates improved oxidative status after dietary supplementation in sheep.

The antimicrobial evidence is likewise mechanistic. Blackberry leaf compounds have inhibited Helicobacter pylori, and ellagic acid derivatives from *R. ulmifolius* have inhibited Staphylococcus aureus biofilm formation in vitro [29,30]. These target organisms and exposure conditions differ fundamentally from rumen fermentation, and nonspecific antimicrobial activity could be beneficial, neutral or detrimental depending on the microbial functions affected.

The defensible interpretation is therefore that antioxidant and antimicrobial profiles justify targeted measurement of rumen ecology and host biomarkers; they are not evidence that supplementation improves immunity, oxidative status or health in sheep.

### 6.3. Antiparasitic Potential and Small-Ruminant Relevance

Gastrointestinal nematodes are a major constraint in sheep systems, particularly under grazing conditions. The direct relevance of *R. ulmifolius* is strengthened by evidence that its extracts show anthelmintic activity against *H. contortus*, a key nematode parasite of small ruminants [14]. This finding is important because it is more directly connected to sheep and goats than many purely human-health phytochemical studies.

Tannin-rich plants can affect gastrointestinal parasites through multiple mechanisms, including direct effects on larvae or adult worms, interference with egg hatching, improved host protein nutrition, and enhanced resilience or immunity [36,37]. However, in vitro anthelmintic activity does not guarantee in vivo parasite control. Ruminal transformation, the dose, feeding duration, parasite species, host immunity and diet quality can alter outcomes. Moreover, excessive tannins may reduce intake or digestibility, which could worsen resilience despite antiparasitic effects.

*R. ulmifolius* should be evaluated, at most, as a possible component of integrated parasite management rather than as an alternative to anthelmintic drugs. Any in vivo claim would require fecal egg counts, larval development, packed cell volume, body condition, growth or milk performance and controlled parasite-challenge studies, with nutritional effects assessed simultaneously.

### 6.4. Bioactive Compounds, Meat Quality and Product Value

Dietary tannins and phenolics can influence product quality in ruminants by affecting fatty acid biohydrogenation, oxidative stability and nitrogen metabolism. A recent meta-analysis of dietary tannins in small ruminants reported effects on nutrient intake, nitrogen-related variables and meat fatty acid profiles, although responses depended on the tannin dose and context [32]. Earlier work also emphasized that tannins can improve some product-quality traits while potentially compromising intake or digestibility if used poorly [11,38].

For *R. ulmifolius*, product-quality effects remain hypothetical because no direct sheep-feeding trial has demonstrated changes in meat fatty acids, oxidative stability, color, sensory traits or shelf-life. These are secondary future endpoints and are relevant only if safety, intake and animal performance are maintained.

### 6.5. From Nutraceutical Claims to Feed-Functional Mechanisms

Pharmacological activity cannot be translated directly into animal-feeding efficacy. Extract studies use solvents, concentrations, exposure times and target organisms that differ from the intact biomass consumed by sheep and transformed through mastication, ruminal fermentation, microbial metabolism and intestinal absorption. The phytochemical profile therefore generates testable feed-functional mechanisms, not a proven nutraceutical claim.

The first plausible mechanism is ruminal protein protection. Condensed tannins can form complexes with dietary proteins, reducing ruminal degradation and potentially increasing post-ruminal amino acid supply when concentrations are moderate. This mechanism is well established for tannin-containing feeds, but it is still untested for *R. ulmifolius* in sheep. Demonstrating it would require measurements of ruminal ammonia–N, soluble protein, microbial protein synthesis, fecal nitrogen, urinary nitrogen and animal nitrogen retention. Without those endpoints, any claim about improved protein use remains speculative.

The second plausible mechanism is antiparasitic support. *R. ulmifolius* extracts have shown activity against *H. contortus*, and the broader literature supports anthelmintic effects of some tannin-containing plants [14,36,37]. However, parasite control in vivo depends on the parasite species, life stage, dose, host immunity, diet quality and grazing management. A robust sheep study would therefore need fecal egg counts, larval development assays, packed cell volume, body condition, clinical signs, pasture contamination and ideally parasite burden at necropsy in a controlled setting. Future research can propose this pathway, but it should not present it as established practice.

The third mechanism is oxidative and inflammatory modulation. Polyphenol-rich plants can alter oxidative status, but this is difficult to translate because biomarkers respond to stress, diet, health status and sample timing. Future trials should include a minimal panel such as total antioxidant capacity, glutathione-related enzymes, malondialdehyde or other lipid peroxidation markers, haptoglobin or acute-phase proteins, and perhaps immune cell phenotyping in more advanced studies. The key point is that a “high antioxidant capacity” in vitro is only meaningful for sheep nutrition if it predicts a measurable host response.

Microbial modulation must also be selective rather than broadly antimicrobial. A desirable response would preserve fibrolysis and overall fermentation while reducing excessive proteolysis, ammonia loss, methanogenesis or pathogen-associated risk. General extract inhibition cannot establish such an outcome.

Dose–response is central: low inclusion may be biologically irrelevant, moderate inclusion may produce useful shifts, and high inclusion may depress intake or digestibility. *R. ulmifolius* can be considered promising only if harvest, processing and the dose preserve any functional signal below the threshold at which tannins, lignin or physical structure reduce performance.

## 7. Antinutritional Constraints and Safety Boundaries

### 7.1. Tannins as a Dose-Dependent Benefit–Risk System

Tannins are the central benefit–risk factor in *R. ulmifolius* (Table 5). Historically, tannins were viewed mainly as antinutritional because they bind proteins, reduce palatability and depress digestibility. Modern ruminant nutrition treats them more precisely: effects depend on the tannin type, dose, molecular structure, protein affinity, basal diet, animal species and adaptation [10,11,33,34]. Low-to-moderate concentrations may reduce excessive ruminal protein degradation and shift nitrogen excretion from urine to feces, while high concentrations can reduce intake and fiber digestion.

This dose-dependent duality is exactly why *R. ulmifolius* should not be recommended without controlled inclusion studies. If harvested biomass has moderate tannin activity and is included at low levels in a mixed diet, it may improve nitrogen-use efficiency or provide functional effects. If mature, tannin-rich and lignified material is included at high levels, it may depress intake and digestibility. The same plant can be beneficial, neutral or harmful depending on how it is used.

Polyethylene-glycol studies are useful because they reveal tannin activity, but PEG is mostly a research tool and not a routine farm solution. A positive PEG response should be interpreted as a warning and an opportunity: a warning because tannins can constrain digestion; an opportunity because controlled tannin activity may be responsible for functional effects. Future work should quantify not only total tannin concentration but also biological activity, because tannins with the same concentration can differ in protein-binding capacity.

### 7.2. Lignification and Physical Limitations

Beyond tannins, lignification is a major constraint. Acid detergent lignin in *R. ulmifolius* increased substantially in summer in the dataset of Laadjal et al. [13]. Lignin reduces microbial access to cellulose and hemicellulose, lowering fiber digestibility and increasing rumen fill (Table 5). In practice, mature cane-rich biomass may behave more like low-quality roughage than functional forage. This is why the plant fraction and harvest timing must be standardized.

Thorns and woody stems also create physical limitations. Sheep may refuse thorny material or selectively consume leaves, producing large refusals and a mismatch between offered and consumed composition. This can confound trials if researchers analyze the offered diet but not refusals. Any in vivo study should measure both offered and refused *R. ulmifolius* fractions to estimate actual intake. Particle size reduction may reduce selectivity, but excessive grinding can alter rumen function and may not remove all physical discomfort.

Processing could partly overcome these limitations. Drying and grinding young shoots could generate a meal suitable for mixing. Pelleting could improve uniformity but may require energy input and binders. Ensiling may soften structure, but phenolic stability and fermentation quality must be evaluated. The optimal processing method will depend on the scale: small farms may use chopping and drying, whereas larger programs could explore pelleted supplements.

### 7.3. Toxicology, Residues and Ecological Biosecurity

No strong evidence currently indicates that *R. ulmifolius* is inherently toxic to ruminants at moderate browse exposure. Nevertheless, the absence of evidence is not proof of safety. Toxicological assessment should consider tannin overload, mineral imbalances, pesticide residues from previously treated stands, fungal contamination during storage, and physical injuries from thorns. Biomass collected from roadsides, contaminated sites or herbicide-treated areas should not be used for feeding.

Ecological biosecurity is equally important. Because *R. ulmifolius* is invasive, feed use must avoid spreading seeds or viable vegetative fragments. Harvesting before fruit ripening, composting or destroying residues, preventing movement of viable canes, and cleaning equipment are essential. Dried or pelleted material may reduce propagation risk, but seed survival after processing should be tested if fruits are included. The review therefore recommends excluding fruits from feed material unless seed viability is demonstrably eliminated.

Regulatory aspects will differ by country. In some jurisdictions, moving invasive plant material may be restricted. In organic or agroecological systems, using local biomass could be attractive because herbicide use is limited, but organic certification may impose additional rules for feed sourcing, contamination and traceability. A future implementation plan should include regulatory review before farm-scale distribution.

### 7.4. Essential Quality Control, Feed Safety and Biosecurity Prerequisites

Quality control is an essential prerequisite because biomass collected from unmanaged invaded sites may contain soil or fecal contamination, herbicide residues, heavy metals, road dust, fungal growth or highly variable moisture (Table 6). Collection-site traceability, exclusion zones, representative residue testing and defined storage standards are required before any animal feeding.

The first biosecurity issue is propagule movement. *R. ulmifolius* spreads through seeds and vegetative structures, so harvesting and transporting fresh material could unintentionally move viable propagules to new areas. For this reason, any feed-use strategy must be designed so that it does not increase invasion. Avoiding ripe fruits in harvested feed, processing material near the collection site, drying or ensiling biomass, and cleaning equipment after harvest are practical safeguards that should be tested and formalized.

The second issue is residues. Plants collected from roadsides, industrial margins, orchards or previously treated areas may contain contaminants that are unacceptable in animal feed. This is especially relevant if sheep products enter human food chains. A practical *Rubus*-feed protocol should therefore define exclusion zones, require traceability of collection sites, and include residue screening when biomass is collected from areas with uncertain management history.

The third issue is microbial and fungal quality. High-moisture chopped material can heat, mold or ferment unpredictably if not used quickly. Dried material can also absorb moisture during storage. Because phenolic-rich plants are sometimes assumed to be self-preserving, this point deserves emphasis: antimicrobial activity in vitro does not eliminate the need for feed hygiene. Future studies should report storage conditions, water activity when possible, visible mold, mycotoxin screening in conserved material and microbial counts in feed batches intended for animal trials.

The fourth issue is physical safety. Thorny material can reduce intake, increase sorting and potentially irritate the oral mucosa if offered as coarse biomass. Chopping, grinding, pelleting or mixing with other ingredients may reduce this risk, but the effect should be measured rather than assumed. Refusals should be separated into leaf, stem and thorn-rich fractions to determine whether animals selectively avoid the most problematic components.

The objective is not to convert invasive biomass into feed at any cost, but to establish a controlled and traceable pathway in which propagule, contaminant, hygiene, physical-safety and nutritional risks are demonstrably lower than the anticipated resource-recovery benefit.

## 8. Rumen Ecology and Microbiome-Centered Hypotheses

### 8.1. The Rumen as the Key Translation Site

The rumen is the biological interface between *R. ulmifolius* chemistry and animal performance. Bacteria, archaea, fungi and protozoa ferment feed into volatile fatty acids, microbial protein and gases, and diet-dependent microbial variation is associated with feed efficiency, nitrogen use, methane and product traits [39,40,41]. Any future claim of functional value would therefore require rumen endpoints linked to intake, digestibility and animal response.

Sheep studies have associated rumen bacterial and archaeal profiles with feed efficiency [42,43], but they did not test *R. ulmifolius*. They provide a rationale for measurement, not evidence that this plant produces a beneficial microbiome shift.

**Figure 2:**
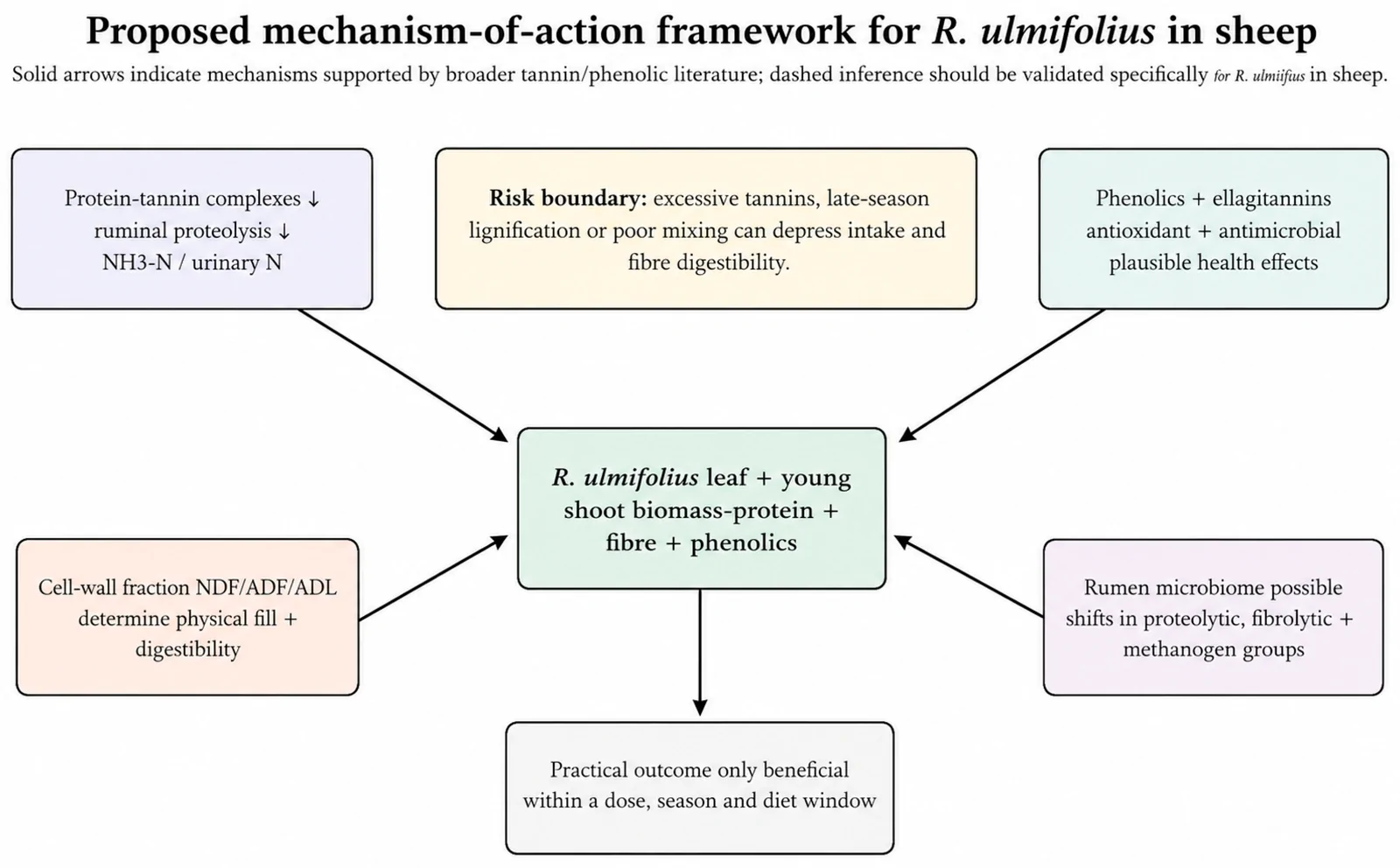
Conceptual mechanism-of-action framework linking *R. ulmifolius* biomass with rumen nitrogen metabolism, fiber digestion, microbial ecology and methane-related pathways. All links are inferred from general tannin or polyphenol evidence and require species-specific validation in sheep.

The central hypothesis is that phenolic and tannin compounds from *R. ulmifolius* could modulate ruminal fermentation by altering substrate availability, protein degradation, microbial enzyme activity, protozoal populations, methanogen-associated hydrogen flow and biohydrogenation pathways. This is plausible based on the tannin literature, but it remains untested specifically for this plant in sheep (Figure 2).

### 8.2. Protein Metabolism and Nitrogen Partitioning

Tannins can bind dietary protein at ruminal pH, reducing microbial proteolysis and ammonia formation. Some protein–tannin complexes dissociate at lower pH in the abomasum or small intestine, potentially increasing amino acid flow post-ruminally. This mechanism is one of the best-established beneficial pathways for moderate condensed tannin intake [11,16,34]. It may also reduce blood urea nitrogen and urinary nitrogen losses, with environmental benefits.

For *R. ulmifolius*, this mechanism is plausible but not quantified. The plant contains tannins and relatively useful crude protein, but the proportion of protein protected versus unavailable is unknown. Excessive binding could reduce total protein digestibility, whereas moderate binding could improve nitrogen-use efficiency. Therefore, future trials should measure ruminal ammonia nitrogen, blood urea nitrogen, urinary nitrogen, fecal nitrogen, microbial protein synthesis and apparent nitrogen digestibility.

An informative design would compare a basal control, low and moderate *R. ulmifolius* inclusion, and a nutrient-matched control without the same tannin exposure. Without protein and fiber matching, responses cannot be attributed confidently to secondary metabolites.

### 8.3. Fiber Digestion, Fibrolytic Microbes and the Risk of Over-Suppression

Tannins can suppress some rumen microbes and enzymes, including fibrolytic activity when concentrations are high. McSweeney et al. emphasized that microbial interactions with tannins have nutritional consequences [44], and Salami et al. showed that hydrolyzable and condensed tannins can affect ruminal fermentation and microbiome composition in lambs [45]. Mannelli et al. also demonstrated that chestnut tannin extract and related phenolics altered rumen liquor microbial community composition in vitro [46].

This matters because any reduction in methane or ammonia is not automatically beneficial. If *R. ulmifolius* reduces methanogenesis by depressing fiber degradation, animal performance may decline and methane intensity per kg of product may not improve. Therefore, future studies should measure methane alongside digestibility, volatile fatty acids and performance. A lower methane output per day can be misleading if dry matter intake or growth also decreases.

The target is selective modulation: lower proteolysis and ammonia, stable or improved volatile-fatty-acid patterns, no major depression of fibrolytic microbes, maintained NDF digestibility and stable intake. This outcome is possible with some tannin sources but remains untested for *R. ulmifolius*.

### 8.4. Methane Mitigation: Hypothesis, Not Marketing Claim

Plant secondary metabolites have been investigated as methane-mitigation tools, but responses vary by compound, dose, animal, diet and method [47,48,49,50,51]. Sheep studies with *Caragana korshinskii* tannin confirm that tannins can alter fermentation, methanogens and metabolomic profiles [52], but they cannot predict the response to *R. ulmifolius*.

For *R. ulmifolius*, the available methane-related evidence is in vitro and context-specific [13]. In vivo assessment should distinguish methane per animal per day, methane yield per kg dry matter intake and methane intensity per kg gain, milk or other product, while measuring digestibility and performance concurrently.

The plant should therefore be described as a candidate for methane screening, not as a methane-reducing feed. A reduction is meaningful only if it is not explained by lower intake or suppressed fiber digestion and if methane intensity improves without a performance penalty.

### 8.5. Biohydrogenation, Fatty Acids and Product-Quality Microbiology

Rumen microbes also control lipid biohydrogenation, which affects the fatty acid profile of meat and milk. Francisco et al. reported relationships between rumen ciliate protozoa and biohydrogenation fatty acid profiles in the rumen and meat of lambs [53]. Tannins can influence ruminal biohydrogenation by affecting microbial populations and enzyme activity, which may alter the deposition of beneficial fatty acids [32,38].

For *R. ulmifolius*, the link to meat quality is an attractive but unproven hypothesis. The appropriate research question is whether low or moderate inclusion modifies rumen protozoa, bacterial taxa involved in biohydrogenation, and the lamb meat fatty acid profile without negative effects on growth. This would require integrated sampling of diet, rumen fluid, feces, blood and meat. Such studies could create a more original paper than a simple digestibility trial.

In a future experimental program, fatty acid endpoints should be secondary unless performance and safety are first established. Product-quality claims are valuable only if the feed is practical and animal productivity is not compromised.

### 8.6. Omics Endpoints That Would Convert the Hypothesis into Mechanistic Science

A major opportunity for increasing novelty is to move beyond conventional feed evaluation and propose a microbiome- and metabolome-informed framework (Table 7). Classical endpoints such as intake, digestibility and weight gain are essential, but they may not reveal why a tannin-containing browse succeeds or fails. *R. ulmifolius* is interesting precisely because its value may emerge through interactions among plant secondary metabolites, rumen microbes, nitrogen metabolism, fatty acid biohydrogenation and host oxidative status. These interactions require more than proximate analysis.

A minimal microbiome study should distinguish bacteria, archaea, protozoa and fungi. Bacterial 16S rRNA sequencing can detect broad shifts in fibrolytic, amylolytic, proteolytic and lactate-utilizing groups. Archaeal profiling is needed because methane production depends on methanogenic communities and hydrogen availability. Protozoa should not be ignored because they contribute to starch and protein turnover, interact with methanogens and participate in lipid biohydrogenation. Anaerobic fungi may also be relevant when woody or lignified substrates are included because they contribute to physical disruption of plant cell walls.

Metagenomics and metatranscriptomics would be stronger than amplicon sequencing when the objective is functional inference. If *R. ulmifolius* modifies methane, ammonia or fiber digestion, the key information is not simply taxonomic abundance but the functional potential and activity of microbial pathways. Genes related to methanogenesis, fibrolysis, proteolysis, amino acid deamination, tannin degradation and phenolic metabolism would be particularly relevant.

The metabolome is equally important. Rumen metabolomics could identify changes in volatile fatty acids, phenolic metabolites, amino acid derivatives, biogenic amines, hydrogen sinks and lipid intermediates. Plasma metabolomics could help determine whether plant-derived metabolites or microbial products are associated with systemic antioxidant or inflammatory responses. Meat metabolomics and fatty acid profiling could be used if the study targets lamb product quality. These endpoints would transform the review from a descriptive feed-resource paper into a mechanistic hypothesis paper.

The strongest design would integrate plant chemistry, rumen fermentation, microbial ecology and host phenotype in the same animals. For example, a study could correlate the tannin activity and phenolic profile of a leaf-enriched *Rubus* supplement with ruminal ammonia–N, methane yield, bacterial and archaeal profiles, plasma oxidative markers, fecal egg count and average daily gain. Such integration would allow researchers to identify response signatures rather than isolated effects. It would also make negative results more informative because a lack of performance response could still be explained by intake limitation, insufficient dose, excessive lignin, microbial adaptation or the absence of bioavailable metabolites.

Experimental reproducibility requires a voucher specimen or equivalent botanical documentation, collection coordinates, phenological stage, leaf:stem ratio, processing method, storage time and batch-level chemistry. This is particularly important because the climate, soil, season, disturbance and genotype can produce substantial chemical plasticity.

## 9. Practical Inclusion Strategies for Sheep Diets

### 9.1. Strategic Use Scenarios

The most realistic practical scenarios are: (i) controlled browsing of invaded margins by sheep or mixed sheep–goat groups; (ii) cut-and-carry feeding of young leaves and shoots; (iii) dried *R. ulmifolius* meal mixed into a basal ration; (iv) pelleted supplements combining *R. ulmifolius* with energy or protein carriers; and (v) extract-based functional additives. Each scenario has different evidence needs, costs and biosecurity risks.

Controlled browsing is operationally simple but difficult to standardize nutritionally. Animals select plant parts, intake is hard to measure, and ecological effects depend on stocking density, timing and repeated access. Cut-and-carry systems allow better control of the plant fraction and inclusion level but require labor and processing. Dried meal or pellets improve ration uniformity and reduce thorn problems but require equipment, drying energy and quality control. Extracts concentrate bioactive compounds but move away from the circular forage concept and require safety evaluation.

For sheep, the safest initial strategy is low-level inclusion of processed young biomass within a mixed diet. This approach minimizes refusal and reduces the risk of excessive tannin load. It also allows experimental control, because actual inclusion can be measured. Direct grazing may be useful for vegetation management, but feed-value claims should be supported by controlled pen or metabolism studies.

### 9.2. Inclusion Rates: What Can and Cannot Be Recommended Now

No universal inclusion rate can currently be recommended for sheep. The available evidence supports only experimental dose categories, because no controlled trial has defined a safe or effective farm dose for *R. ulmifolius*.

Initial studies could compare approximately 2.5–5% and 7.5–10% of diet dry matter, with a higher exploratory level of 15% or more only under intensive monitoring (Table 8). These are research categories rather than feeding recommendations and must be adjusted to tannin activity, lignin, basal diet, animal class and ethics approval. A polyethylene-glycol treatment can be used in vitro or in selected mechanistic designs to help identify tannin-mediated effects.

In trials, *R. ulmifolius* should be introduced gradually. Researchers should monitor dry matter intake, refusals, fecal consistency, body weight, body condition, rumination behavior and clinical signs. If intake declines, feces become abnormal, or digestibility drops, inclusion should be reduced. Feeding studies should also include chemical analysis of each batch because seasonal variability makes a single value unreliable.

### 9.3. Processing and Preservation Options

Processing should be chosen according to the farm scale and research objective (Table 9). Chopping is the simplest method and may reduce the physical barrier created by thorns, but it does not standardize nutrient composition if mature stems are included. Drying stabilizes biomass and allows storage, but high temperatures may alter phenolic compounds. Grinding improves mixing but can increase dust and reduce physically effective fiber. Pelleting improves handling and intake uniformity but adds cost. Ensiling could be explored if moisture and fermentable carbohydrate conditions are adequate, but tannins may affect fermentation microorganisms.

An attractive product concept is a dried young-shoot meal harvested after cutting-induced regrowth. Such material could have a higher leaf-to-stem ratio and more consistent quality than biomass from unmanaged thickets. A second concept is a mixed pellet containing *R. ulmifolius* meal, a low-tannin forage base and an energy carrier. A third is a targeted functional additive based on standardized phenolic extracts, but this would require more regulatory and safety work.

Quality control should include moisture, crude protein, NDF, ADF, ADL, ash, tannin activity, mold inspection and the absence of herbicide residues. If the material is commercialized or distributed, traceability should document the harvest location, date, phenological stage, processing method and storage conditions.

### 9.4. Animal Category and Production Objective

The animal class and physiological stage must be explicit because maintenance ewes, growing lambs, pregnant or lactating ewes and dairy sheep differ in nutrient demand and tolerance of variable feed quality. A cautious sequence would begin with acceptability and safety in adult maintenance animals, proceed to growing lambs, and evaluate lactating or pregnant ewes only after inclusion boundaries are defined.

For growing lambs, the key endpoints are dry matter intake, average daily gain, feed conversion, apparent digestibility, rumen fermentation, methane intensity and meat quality. For lactating ewes, endpoints include milk yield, milk composition, body condition, lamb growth and metabolic indicators. For parasite-challenged grazing sheep, fecal egg counts and resilience indicators become central. The feeding objective determines whether *R. ulmifolius* is evaluated as a nutrient source, functional additive, parasite-management support or methane-mitigation candidate.

Farm implementation, if later justified, should begin with animals at low nutritional risk, gradual introduction and close monitoring. *R. ulmifolius* should not become the dominant roughage unless controlled evidence supports that use.

### 9.5. A Decision Framework for Experimental and On-Farm Inclusion

Because direct sheep trials are scarce, responsible use requires a decision framework rather than a universal prescription (Table 10). Biomass should first pass site traceability, contaminant, fruit/propagule and leaf:stem criteria; the objective should then be defined as nutritional supplementation, functional testing or biomass management; and processing and storage capacity must be confirmed before feeding.

Experimental diets should begin with low and moderate inclusion in balanced rations, followed by a higher level only when safety and intake are maintained. All levels must be labeled as research doses until direct sheep data establish validated thresholds.

The decision framework also needs an exit criterion. If the supplement reduces dry matter intake, increases refusals, depresses fiber digestibility, produces abnormal fecal consistency, or reduces average daily gain, the inclusion level is too high or the processing form is unsuitable. Conversely, a promising response would include maintained or improved intake, stable fiber digestion, reduced ruminal ammonia–N without loss of microbial protein synthesis, lower methane per unit of digestible organic matter, improved parasite indicators or improved oxidative status. These endpoints should be framed as measurable decision points rather than vague benefits.

In practical systems, *R. ulmifolius* may be most useful where it is already present and where removal is already planned. This avoids creating incentives to cultivate or spread an invasive plant. The supplement should be viewed as a by-product of control, not as a crop. This is an essential ethical and ecological boundary: valorization should reduce the net burden of invasion, not create a new production chain that maintains invasive stands for feed supply.

This decision tree converts broad benefits into measurable advance or exit criteria and preserves the distinction between an interesting invasive biomass and a usable, validated feed ingredient.

## 10. Circular Bioeconomy and Sustainability Dimensions

### 10.1. Converting a Control Cost into a Resource Flow

The circular bioeconomy value of *R. ulmifolius* lies in converting a recurrent management cost into a resource flow. In invaded landscapes, biomass removal already requires labor, fuel, herbicides or grazing. If part of the removed biomass is usable as feed, the cost–benefit equation changes. This is not because *R. ulmifolius* becomes a high-value crop, but because waste biomass acquires a secondary function. Such logic is particularly attractive in low-input sheep systems where feed costs and seasonal scarcity are major constraints.

However, circularity must be quantified. A true sustainability analysis should compare business-as-usual control with valorization scenarios. Variables should include fuel consumption, labor, machinery depreciation, herbicide reduction, biomass yield, processing cost, storage loss, feed replacement value, animal performance and ecological outcomes. Without this quantitative layer, sustainability claims remain rhetorical.

Official emission factors indicate that diesel combustion produces substantial CO2 emissions per unit fuel, and machinery operations for brush control require energy inputs. Therefore, avoiding repeated mechanical passes or extracting feed value from a single pass could improve resource efficiency. Still, processing, drying and transport also consume energy. The net balance must be calculated rather than assumed.

### 10.2. Organic, Agroecological and Smallholder Relevance

Organic and agroecological systems may be particularly interested in *R. ulmifolius* valorization because herbicide options are limited and consumers often value local resource use. Lorenz and Lal discussed environmental aspects of organic agriculture, and the broader sustainability literature emphasizes reducing external inputs where feasible [54]. In this context, harvesting invasive biomass for local feeding could align with agroecological principles, provided that contamination and biosecurity risks are managed.

Smallholder feasibility depends on local conditions. Chopping young biomass and mixing it with hay may be realistic where removal is already required and transport is short, whereas drying, pelleting and centralized quality testing may require cooperatives, municipalities or service providers. These remain implementation scenarios rather than established systems.

A specific opportunity exists in regions where *R. ulmifolius* is abundant near sheep farms. Proximity matters because transport can erase sustainability benefits. The most defensible model is local use of local biomass, with minimal transport and clear no-spread protocols.

### 10.3. Ecological Safeguards and Ethical Framing

Valorization can create perverse incentives if landholders retain or disseminate an invasive plant for feed. The ethical boundary is therefore explicit: *R. ulmifolius* should not be cultivated, planted or spread; only biomass from existing stands under control or containment may enter the proposed pathway.

No-spread safeguards should be treated as non-negotiable. Feed-chain protocols should prevent movement of viable seeds or stems, avoid harvest during fruiting unless seed viability is destroyed, and dispose of residues safely. If fruits are included in harvested material, processing methods must be validated for seed inactivation. If stems are transported fresh, containment is required. These details are not merely operational; they are central to the ecological acceptability of the strategy.

### 10.4. Preliminary Life-Cycle Thinking: What Should Be Counted

A circular-bioeconomy claim is only defensible if the system boundary is clear. For *R. ulmifolius*, the boundary should include current control practices, harvesting operations, transport, processing, storage losses, feed replacement value, animal responses and ecological outcomes (Table 11). If only the feed value is counted, the analysis will overestimate benefits. If only the control cost is counted, it will ignore nutritional uncertainty. A credible framework must compare “control and disposal” with “control plus feed valorization”, not with an unrealistic scenario in which *Rubus* biomass appears without collection or processing cost.

The first inventory category is avoided control burden. Mechanical cutting, shredding, herbicide application and repeated clearing have direct costs and environmental impacts. However, feed valorization does not eliminate all control costs; harvesting and processing also require labor and energy. Therefore, the benefit is not simply the diesel or herbicide avoided, but the net difference between the current management pathway and a valorization pathway that still requires safe biomass handling.

The second category is feed substitution. If *R. ulmifolius* replaces part of a purchased roughage or protein supplement without reducing animal performance, it may reduce feed costs and upstream impacts. If it reduces intake or digestibility, the system may require compensatory feed and lose its sustainability advantage. Thus, feed substitution must be performance-adjusted. The correct unit is not kilograms of *Rubus* biomass, but kilograms of digestible nutrient supplied, animal product maintained, or control biomass valorized per unit of environmental burden.

The third category is ecological consequence. Harvesting invasive biomass may reduce local thicket density, improve access, lower propagule pressure or support restoration, but it may also disturb soil, spread fragments, remove habitat used by wildlife or create incentives to maintain the invasive stand. These opposing possibilities mean that ecological indicators should be included in future pilots. A practical set could include standing biomass before and after harvest, regrowth rate, fruiting reduction, native vegetation recovery and evidence of propagule dispersal during handling.

The fourth category is animal efficiency. In ruminant systems, environmental intensity is strongly shaped by feed conversion, methane production and nitrogen losses. A *Rubus* supplement that modestly reduces methane but depresses growth could be environmentally neutral or negative per kilogram of lamb. Conversely, a supplement that maintains growth while reducing purchased feed or improving nitrogen partitioning could provide a more credible benefit. This is why life-cycle thinking must be connected to animal performance rather than treated as a separate narrative.

A full life-cycle assessment will require local emission factors, transport distances, machinery and processing data, allocation rules and measured animal performance. Until those data exist, sustainability claims should be limited to a structured inventory comparing control-and-disposal with control-plus-valorization.

### 10.5. Adoption, Governance and Producer Behavior

Even if *R. ulmifolius* proves nutritionally useful, adoption will depend on labor, equipment, safety and perceived reliability. Producers are unlikely to harvest thorny shrubs if the process is slow, dangerous or nutritionally uncertain. For this reason, the most realistic adoption pathway may involve organized biomass management rather than individual opportunistic cutting. Cooperatives, municipalities, restoration projects or farm-service providers could integrate shrub removal with feed processing if the product meets safety and nutritional standards.

Governance is also necessary to avoid perverse incentives. If an invasive plant acquires feed value, landholders might tolerate dense stands or even move biomass in ways that increase spread. A responsible valorization model should therefore be linked to control objectives, not cultivation objectives. Certification or local guidelines could specify that material must come from removal or containment activities, that fruiting material should be avoided or processed to eliminate seed viability, and that residues must be disposed of safely.

Producer adoption also requires a simple quality language. Laboratory values such as NDF, ADL and condensed tannins are essential for research, but farms need practical decision points: harvest before heavy lignification, favor leaf-rich material, avoid contaminated areas, chop or process before feeding, introduce gradually, combine with conventional forage and monitor refusals. The review can bridge these levels by translating analytical findings into operational rules while clearly stating that final thresholds require direct validation.

**Figure 3:**
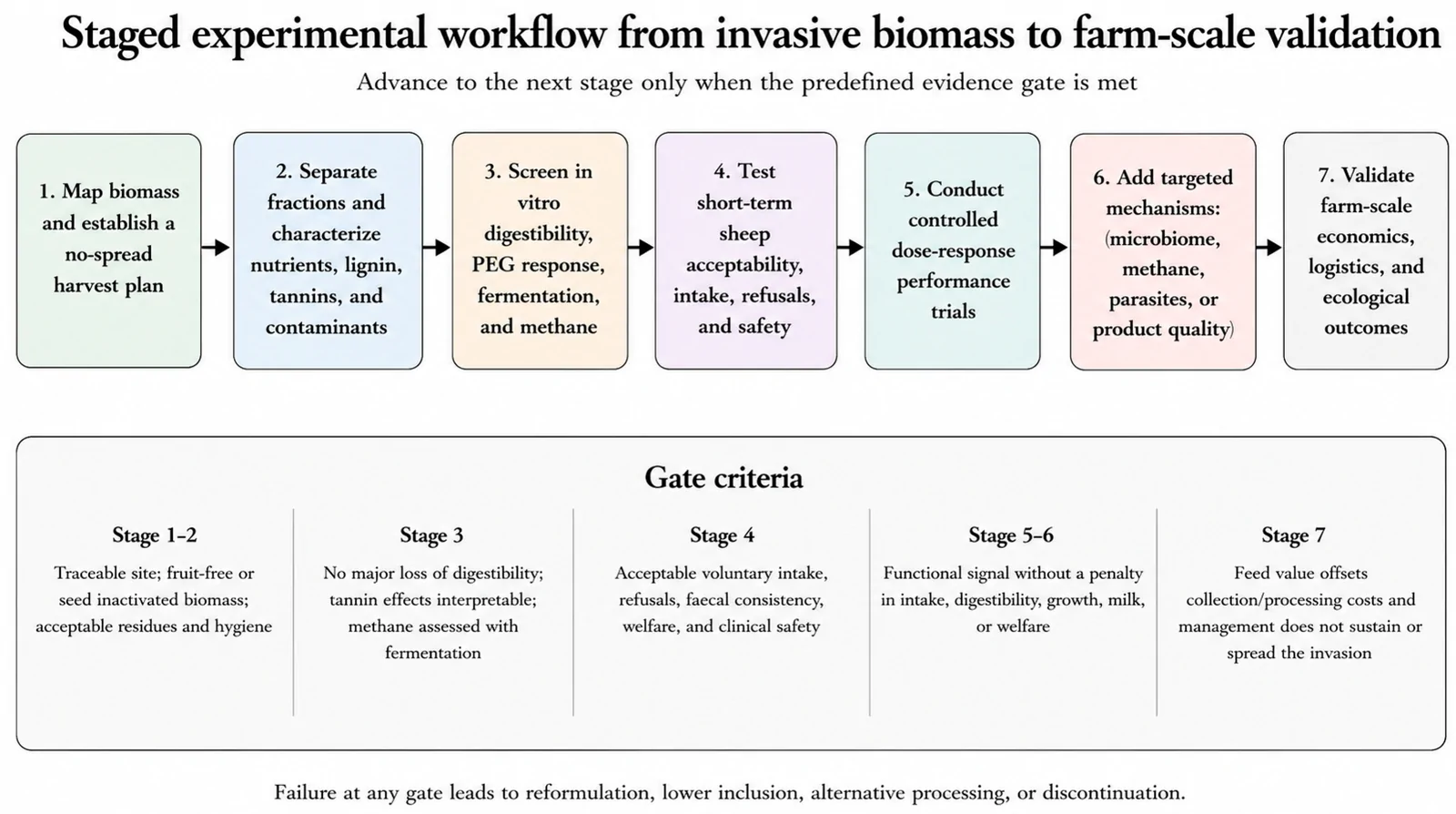
Proposed staged workflow from biomass collection to farm-scale validation. The sequence proceeds through biomass mapping and no-spread harvest, chemical and safety characterization, in vitro rumen screening, short-term sheep acceptability and safety, dose–response performance trials, targeted mechanistic modules, and farm-scale techno-economic and ecological validation. Advancement requires predefined evidence gates.

Finally, economic analysis should include labor opportunity cost. A free invasive plant is not free if harvesting takes time, damages equipment or requires transport. The value proposition becomes stronger when *Rubus* removal is already funded or necessary, when biomass is close to the animals, when equipment is available, and when the resulting supplement replaces purchased feed without reducing performance (Figure 3).

## 11. Staged Validation Framework from Biomass Collection to Farm-Scale Evaluation

### 11.1. Stage 1: Biomass Mapping and Harvest Standardization

The first research stage should quantify biomass availability and define harvest standards. Studies should map invaded areas, estimate seasonal biomass yield, separate plant fractions, record phenological stage and assess regrowth after cutting. Importantly, biomass estimates should distinguish total above-ground biomass from usable feed biomass. A thicket may produce large biomass, but only a fraction may be suitable for sheep after excluding mature woody stems, contaminated material and fruiting structures.

Harvest standardization should include the date, regrowth age, cutting height, fraction retained, drying method and storage. Without standardization, nutritional results will be irreproducible. Future papers should also include photographs or detailed descriptions of harvested material. This will help readers understand whether the feed tested was leaf-rich, shoot-rich or stem-rich.

An ecological sub-study should examine whether repeated harvest reduces cover, increases native vegetation access or changes regrowth dynamics. This would connect the feed study with invasive management and increase the originality of the research.

### 11.2. Stage 2: Analytical Characterization

The minimum analytical profile should include dry matter, organic matter, crude protein, ether extract, ash, NDF, ADF, ADL, minerals, total phenolics, condensed tannins, hydrolyzable tannins or ellagitannin markers when feasible, antioxidant capacity, protein-precipitating capacity and microbial contamination [24].

Because *R. ulmifolius* chemistry varies seasonally, analysis should be repeated across seasons and plant fractions. At a minimum, spring, summer, autumn and winter samples should be collected from multiple sites. Multi-site sampling is important because the soil, climate, shade and disturbance may affect composition. A single-site dataset is useful but insufficient for general recommendations.

Analytical results should be linked to practical thresholds. For example, high ADL would discourage use as high-inclusion roughage, whereas high protein with moderate tannin activity might justify low-to-moderate supplementation. The goal is not to produce descriptive chemistry but to define decision rules.

### 11.3. Stage 3: In Vitro Rumen Fermentation and Methane Screening

Before in vivo trials, in vitro rumen fermentation can screen inclusion levels and identify risk. Batch culture or gas-production systems should compare *R. ulmifolius* fractions and processed forms. Endpoints should include gas kinetics, methane, pH, ammonia nitrogen, volatile fatty acids, in vitro dry matter digestibility, in vitro organic matter digestibility and PEG response. Rumen inoculum should preferably come from sheep adapted to forage-based diets.

PEG inclusion is particularly useful because it distinguishes tannin-mediated effects from other plant characteristics. If PEG increases gas or digestibility substantially, tannins are constraining fermentation. If PEG increases methane, tannins may be contributing to methane suppression. However, PEG responses must be interpreted together with digestibility; a methane decrease driven by suppressed fermentation is not necessarily beneficial.

In vitro comparisons should include a conventional forage control and a known tannin source. A factorial structure that tests the season, plant fraction, processing form and inclusion level would provide the clearest basis for selecting material for animal trials.

### 11.4. Stage 4: Controlled Sheep Trials

Controlled sheep trials are essential. The first trial should be a palatability and short-term intake study using low and moderate inclusion levels of processed young biomass. The second should be a digestibility and nitrogen-balance trial. The third should evaluate performance in growing lambs or lactating ewes. Only after these stages should parasite or methane claims be tested at a larger scale.

Experimental diets should be formulated to avoid confounding. If *R. ulmifolius* increases crude protein, a protein-matched control is needed. If it increases fiber, a fiber-matched control may also be appropriate. Diets should be isoenergetic when possible. Actual intake should be calculated from offered feed minus refusals, and refusals should be chemically analyzed because sheep may selectively avoid stems.

In vivo endpoints should include dry matter intake, apparent digestibility, rumen pH, ammonia nitrogen, volatile fatty acids, blood urea nitrogen, oxidative biomarkers, fecal egg counts where relevant, methane, weight gain or milk yield, body condition and welfare indicators. Microbiome endpoints should include bacteria, archaea and possibly protozoa and fungi. A combined microbiome–metabolome approach would provide the strongest mechanistic insight.

### 11.5. Stage 5: Farm Pilots and Techno-Economic Assessment

After controlled trials, farm pilots should evaluate feasibility. Variables should include harvest labor, machinery, drying or processing cost, storage stability, acceptance by farmers, animal response under real conditions and ecological outcomes. Economic analysis should compare the feed replacement value with the cost of collection and processing. A feed ingredient that works biologically but is too expensive or difficult to handle will not be adopted.

Farm pilots should also evaluate social acceptability. Producers may be skeptical of feeding their animals a weed, while conservation stakeholders may worry about propagation. Clear communication and biosecurity protocols are essential. Demonstration trials should emphasize that the goal is not to cultivate *R. ulmifolius* but to valorize biomass removed during control.

A strong final research product would be a decision-support tool indicating when *R. ulmifolius* biomass is suitable for feeding: the acceptable season, plant fraction, tannin range, lignin threshold, processing method, animal category and maximum inclusion. Such a tool would represent a tangible output beyond the review (Table 12).

### 11.6. Experimental Design Standards for Future Sheep Trials

Controlled trials should define the harvested fraction, analyze each feed batch, formulate isoenergetic and isonitrogenous diets where possible, include at least two inclusion levels, measure refusals and digestibility, and connect animal responses with rumen and host biomarkers (Table 13). Body weight alone is insufficient to determine whether a tannin-rich browse is safe or functionally useful.

The first design issue is the control diet. If *Rubus* supplementation changes both nutrient supply and secondary-metabolite exposure, interpretation becomes difficult. One control should match the basal diet without *Rubus*. A stronger design could include a nutrient-matched control using a conventional forage or protein source, and a tannin-control treatment using polyethylene glycol in a subset or in vitro assay to test whether observed effects are tannin-mediated. Polyethylene glycol is not necessarily a field solution, but it remains valuable mechanistically because it helps separate tannin effects from other plant properties.

The second design issue is adaptation. Rumen microbes and animals can adapt to tannin-containing feeds over time. Short trials may overestimate negative palatability effects or miss longer-term microbial adaptation. Conversely, very long trials without intermediate sampling may miss early disturbances. A balanced design should include an adaptation phase, repeated sampling and clear stopping criteria. Intake should be measured daily during adaptation because refusal patterns often reveal practical failure before performance differences appear.

The third issue is the animal category. Maintenance ewes, growing lambs and lactating ewes differ in nutrient demand and tolerance for variable feed quality. Early studies should probably begin with maintenance or growing animals under controlled conditions before moving to lactation or reproduction. If lamb growth or meat quality is the objective, carcass traits and fatty acid composition should be included. If ewe resilience during feed gaps is the objective, body condition, reproductive performance and health indicators become more relevant.

Because composition varies by season and site, trials should use a well-characterized composite batch or include the harvest batch as an explicit factor. Batch-level chemistry and archived samples are needed to determine whether a response reflects the species, the harvest environment, the processing method or a specific chemical profile.

A final recommendation is preregistration or at least a clearly stated analysis plan for animal trials. This is not yet routine in all animal nutrition studies, but it would improve transparency when multiple endpoints are measured. Because functional-feed studies can easily become selective reporting exercises, predefined primary outcomes such as dry matter intake, digestibility, methane yield, fecal egg count or average daily gain would strengthen credibility.

## 12. Testable Hypotheses for Future Studies

Six testable hypotheses are summarized in Table 14: early regrowth may provide a better protein-to-lignin balance than mature biomass; moderate inclusion may alter nitrogen partitioning without depressing intake or fiber digestion; phenolics may modulate rumen microbial function in a dose-dependent manner; methane yield may decrease only when digestibility is preserved; parasite resilience may improve only if in vivo outcomes accompany adequate nutrition; and repeated biosecure harvest may support both feed standardization and invasive shrub management. Each hypothesis remains conditional and requires the specific critical endpoints shown in the table.

## 13. Conclusions

Current evidence supports *R. ulmifolius* as an invasive biomass with potentially useful young leaves and shoots, not as a validated sheep supplement. Species-specific chemical data show seasonal and fraction-dependent variation, while goat browsing, extract bioactivity and the general tannin literature provide only indirect support for hypotheses concerning intake, nitrogen use, methane, microbiome modulation, parasite resilience, oxidative status or product quality. Major constraints are lignification, the tannin dose, thorns and selective refusal, processing effects, contaminants and the risk of propagule spread.

Evaluation should therefore proceed through a staged, no-spread framework that links fraction-specific characterization and in vitro screening with short-term sheep safety, controlled dose–response trials and farm-scale economic and ecological validation. Only a traceable product that maintains intake, digestibility, welfare and performance while producing a reproducible animal-level benefit could ultimately be considered a functional browse supplement.

## Figures and Tables

**Table 1:** Outcome-specific evidence hierarchy for evaluating *R. ulmifolius* as a candidate sheep supplement.

Outcome Domain	Best Available *R. ulmifolius* Evidence	Current Evidence Level	Permitted Interpretation
Nutrient composition and seasonality	Species-specific chemical datasets, including historical Chilean and modern seasonal analyses [12,13]	Moderate for composition; geographically limited	Supports fraction- and season-specific screening, not a universal feed value
Voluntary intake and palatability	Goat browsing observations; no controlled sheep intake study	Low and indirect	Shows browse exposure in goats only; cannot define sheep intake or dose
Digestibility and tannin activity	In vitro digestibility, gas and polyethylene-glycol responses [13] plus the general tannin literature	Moderate mechanistic evidence	Identifies benefit–risk mechanisms; sheep digestibility trials remain necessary
Methane-related effects	In vitro gas/methane data and evidence from other tannin sources	Low and indirect	Justifies screening only; no claim of reduced methane in sheep
Parasite control	Species-specific in vitro activity against *H. contortus* [14]	Low-to-moderate mechanistic evidence	Supports an integrated parasite-management hypothesis; in vivo efficacy unproven
Microbiome and rumen modulation	General tannin/polyphenol studies; no *R. ulmifolius* sheep microbiome trial	Indirect	Hypothesis only and must be linked to fermentation, digestibility and performance
Animal performance and product quality	No controlled *R. ulmifolius* sheep-feeding evidence	Very low or absent	No established claims for growth, milk, meat, oxidative status or welfare
Farm feasibility and biosecurity	Invasion management, processing and circularity literature	Implementation evidence	Supports a no-spread and cost-assessment framework; local pilots are required

**Table 2:** Seasonal chemical composition of *R. ulmifolius* biomass in a single Algerian dataset [13]. Values are expressed as % dry matter, except DM as % fresh matter.

Season	DM	Ash	Crude Protein	NDF	ADF	ADL	Interpretation
Autumn	31.58 ± 0.71	5.70 ± 0.05	15.24 ± 0.25	37.84 ± 0.37	20.38 ± 0.14	6.19 ± 0.53	Moderate CP and moderate fiber; potentially useful if mixed with basal roughage
Winter	30.37 ± 5.52	5.35 ± 0.04	16.38 ± 0.11	32.49 ± 0.19	18.14 ± 0.93	6.28 ± 0.56	Relatively favorable fiber profile, but field accessibility may be limiting
Spring	22.80 ± 1.36	7.19 ± 0.04	18.88 ± 0.52	34.48 ± 0.37	19.48 ± 0.29	5.11 ± 0.77	Best reported protein–lignin balance; priority harvest window
Summer	31.63 ± 0.80	6.08 ± 0.16	14.81 ± 0.09	45.23 ± 2.92	27.63 ± 0.12	12.97 ± 1.94	Higher lignification; risk of lower digestibility and intake

Abbreviations: DM, dry matter; NDF, neutral detergent fiber; ADF, acid detergent fiber; ADL, acid detergent lignin. Values adapted from Laadjal et al. (2023) [13] and interpreted in the context of ruminant nutrition.

**Table 3:** Fraction- and process-specific interpretation of *R. ulmifolius* as a potential sheep-feed resource.

Fraction or Product	Expected Advantage	Main Limitation	Minimum Characterization Needed
Fresh leaves and young shoots	Higher expected nutritive value and lower lignification than mature canes	Moisture, thorns, selectivity and rapid deterioration after harvest	DM, CP, NDF, ADF, ADL, tannins, total phenolics, in vitro digestibility
Leaf-enriched dried meal	More stable ingredient for ration formulation and experimental dosing	Drying may alter phenolic extractability and antioxidant activity	Particle size, storage stability, phenolic profile, microbial contamination
Chopped mixed biomass	Simpler field use and lower processing cost	Variable leaf:stem ratio and high physical heterogeneity	Leaf:stem ratio, fiber fractions, refusal rate and animal sorting
Pelleted blend with other forages	Improved handling, dose control and lower selectivity	Potential heat effects on bioactive compounds and higher processing cost	Pellet durability, intake, tannin activity after pelleting, digestibility
Ensiled *Rubus*-containing mixture	Seasonal conservation and possible softening of thorny material	Unknown fermentation quality and risk of poor ensiling from woody biomass	pH, lactic/acetic acids, ammonia–N, molds, mycotoxins, aerobic stability

**Table 4:** Major bioactive compound classes reported for *R. ulmifolius* or closely related wild Rubus leaves and the current evidence relevant to sheep nutrition.

Major Compound or Class	Relevant Plant Fraction	Reported Biological Activity	Relevance to Sheep Nutrition/Evidence Level
Ellagitannins and ellagic acid derivatives	Leaves and aerial parts	Antioxidant, antimicrobial and antibiofilm activity [23,29,30]	Mechanistic plausibility only; oral dose, rumen fate and animal response are unknown
Flavonols, including quercetin and kaempferol derivatives	Leaves and aerial parts	Antioxidant activity [23,31]	Indirect chemical evidence; no demonstrated sheep outcome
Phenolic acids	Leaves and aerial parts	Antioxidant and antimicrobial activity [23,31]	May contribute to phenolic exposure, but functional feeding effects are unproven
Condensed and hydrolyzable tannins	Leaves and young shoots	Protein binding and dose-dependent effects on rumen N, digestibility and methane in other feeds [10,11,32,33,34]	Strong general mechanism; *R. ulmifolius*-specific dose and benefit–risk threshold are unknown
Extractable polyphenols active against *H. contortus*	Leaf and fruit extracts	Ovicidal and adult-worm activity in vitro [14]	Species-specific in vitro evidence; integrated parasite-management relevance requires in vivo feeding trials
Anthocyanins	Fruits	Antioxidant and antimicrobial activity [22,35]	Contextual species chemistry only; low feed relevance and fruit should be excluded unless seed viability is eliminated

**Table 5:** Principal benefits, risks and mitigation strategies associated with *R. ulmifolius* supplementation.

Dimension	Potential Benefit	Main Risk	Mitigation or Research Need
Nutrient supply	Moderate-to-high CP in young material; additional roughage source	Actual intake may be low if thorny or lignified	Use young leaves/shoots; measure refusals; process before feeding
Tannins	May reduce ruminal proteolysis, NH3-N and urinary N; possible parasite effects	Excessive tannins may reduce palatability and digestibility	Dose–response trials; tannin assays; PEG diagnostics in vitro
Fiber	Can contribute physically effective fiber if properly processed	High ADL in mature material reduces digestibility	Harvest early; avoid mature cane-rich biomass
Microbiome	Possible modulation of proteolytic microbes, protozoa and methanogens	Unwanted depression of fibrolytic bacteria	16S/ITS/archaea sequencing plus fermentation endpoints
Methane	Tannin-rich feeds may reduce CH4 under some conditions	Lower digestibility can offset benefits per unit product	Measure CH4 per animal and per kg gain or product
Parasites	Extracts show anti-*H. contortus* activity	In vitro activity may not translate in vivo	Controlled parasite trials with fecal egg counts and health endpoints
Biosecurity	Uses biomass from invasive-plant control	Spread via seeds/canes if mishandled	No-spread harvest protocol; exclude fruit; process biomass

**Table 6:** Feed-safety and biosecurity framework required before *R. ulmifolius* can be recommended as a practical sheep supplement.

Risk Domain	Specific Concern	Recommended Control	Research Endpoint
Invasion biosecurity	Movement of seeds or viable vegetative fragments	Avoid ripe fruit, process near harvest site, clean equipment	Seed viability after processing; regrowth from residues
Chemical residues	Herbicides, heavy metals, road dust or industrial contaminants	Use traceable collection zones and exclusion criteria	Residue screening in representative batches
Feed hygiene	Mold, mycotoxins or uncontrolled fermentation	Drying, ensiling validation, protected storage	Water activity, mold score, mycotoxin panel, aerobic stability
Physical safety	Thorns, woody stems and particle heterogeneity	Chopping, grinding, pelleting or leaf enrichment	Refusal composition, oral lesions, intake pattern
Nutritional imbalance	Excess tannins or lignified fiber	Moderate inclusion and mixed diets	DMI, digestibility, rumen NH3-N, fecal N, performance

**Table 7:** Multi-layer endpoint framework for mechanistic studies of *R. ulmifolius* supplementation in sheep.

Layer	Suggested Endpoints	Why It Matters for the *Rubus* Hypothesis
Plant chemistry	CP, NDF, ADF, ADL, soluble protein, CT, HT, total phenolics, flavonoids, antioxidant capacity	Defines the actual biochemical exposure rather than relying on the plant name
Rumen fermentation	pH, NH3-N, total gas, CH4, VFA profile, IVDMD, IVOMD	Tests whether tannins redirect fermentation without suppressing digestibility
Microbiome	16S/ITS, archaeal markers, protozoal counts, metagenomics where feasible	Links fermentation changes to bacteria, archaea, fungi and protozoa
Host metabolism	Nitrogen balance, oxidative markers, acute-phase proteins, blood metabolites	Determines whether rumen effects translate into host physiology
Animal performance	DMI, refusals, ADG, body condition, wool or milk where relevant	Determines whether functional effects are useful in production
Product quality	Meat fatty acids, oxidative stability, sensory traits in lamb studies	Tests whether rumen lipid metabolism produces market-relevant outcomes

**Table 8:** Experimental dose categories for controlled sheep trials; these are not farm feeding recommendations.

Category	Approximate Inclusion (% Diet DM)	Primary Purpose	Required Monitoring	Interpretation
Control	0	Baseline diet	DMI, digestibility, rumen and performance endpoints	Reference response
Low	2.5–5	Acceptability and early signals	Refusals, fecal score, rumen NH3-N and VFA	Initial research range
Moderate	7.5–10	Nutritional and tannin dose–response	DMI, digestibility, N balance, microbiome and methane	Key mechanistic range
High exploratory	>=15	Upper tolerance boundary	Full welfare, intake and digestibility monitoring	Controlled research only; not a farm recommendation

**Table 9:** Processing options for *R. ulmifolius* biomass and their expected advantages and limitations.

Processing Option	Advantages	Limitations	Best Research Use
Fresh controlled browsing	Low equipment demand; integrates vegetation management	Intake difficult to quantify; selective browsing; spread risk if fruiting	Field ecology and behavior studies
Fresh chopped biomass	Simple cut-and-carry approach; partial reduction of thorn barrier	Short shelf-life; refusals possible; variable composition	Pilot palatability trials
Dried meal	Stable, mixable and easier to dose	Drying costs; phenolic changes possible	Controlled dose–response trials
Pellet	Uniform intake; easier transport; lower selectivity	Higher processing cost; may require binders	Product-development studies
Silage/co-silage	Potential preservation for seasonal use	Unknown fermentation quality; tannins may inhibit fermentation	Exploratory conservation trials
Phenolic extract	Standardized bioactive dose	No longer whole-biomass circular feed; higher regulation	Mechanistic rumen or parasite studies

**Table 10:** Decision framework for responsible experimental or farm-level use of *R. ulmifolius* biomass in sheep systems.

Decision Step	Question	Favorable Answer	Unfavorable Answer
Site selection	Is the biomass from an uncontaminated and traceable area?	Proceed to harvest planning	Do not use as feed; manage as waste/control biomass
Plant stage	Is the material leaf- or young-shoot-enriched and mostly fruit-free?	Proceed to processing	Avoid feed use or separate unsuitable fractions
Processing capacity	Can the farm chop, dry, ensile or mix the material safely?	Use controlled small batches	Avoid direct feeding of coarse thorny biomass
Diet objective	Is *Rubus* used as supplement rather than replacement?	Test moderate inclusion in mixed ration	Do not rely on it as sole forage
Animal monitoring	Are intake, refusals and health indicators acceptable?	Continue and collect performance data	Reduce inclusion or discontinue
Ecological outcome	Does use reduce standing invasive biomass without spreading propagules?	Valorization is defensible	Revise protocol; avoid perverse incentives

**Table 11:** Minimum inventory for a preliminary life-cycle and techno-economic assessment of *R. ulmifolius* valorization.

Life-Cycle Domain	Variables to Record	Interpretation for Circularity
Baseline control pathway	Mechanical hours, fuel, herbicide, labor, disposal method, repeated interventions	Defines the cost and impact that valorization could partially offset
Harvest and processing	Labor, fuel/electricity, distance, equipment, drying or pelleting cost, storage losses	Prevents overestimating benefits by ignoring processing burdens
Feed substitution	Nutrient composition, digestibility, inclusion level, replaced feed, feed price	Determines whether *Rubus* supplies useful nutrients or merely dilutes the ration
Animal response	DMI, ADG, methane, nitrogen balance, health indicators	Converts feed use into performance-adjusted sustainability
Ecological outcome	Standing biomass reduction, regrowth, fruiting, native recovery, propagule risk	Ensures feed use contributes to management rather than spread
Economic adoption	Net cost per tonne DM, cost per animal/day, labor acceptability, equipment needs	Determines whether the pathway is realistic for producers

**Table 12:** Minimum dataset recommended for future *R. ulmifolius* sheep-feeding studies.

Dataset Component	Specific Variables	Why It Is Needed
Plant identity and harvest	Location, season, phenology, plant fraction, regrowth age, presence/absence of fruit	Allows reproducibility and biosecurity assessment
Chemical composition	DM, OM, CP, EE, ash, NDF, ADF, ADL, minerals	Defines basal nutritive value and fiber constraints
Phytochemistry	Total phenolics, condensed tannins, hydrolyzable tannins/ellagitannin markers, protein-precipitating capacity	Defines functional and antinutritional potential
Processing	Fresh/chopped/dried/ground/pelleted/silage; drying temperature; particle size	Explains palatability, storage and phenolic stability
Intake and digestibility	Offered feed, refusals, DMI, apparent digestibility, fecal output	Determines whether animals actually consume and utilize the biomass
Rumen fermentation	pH, NH3-N, VFA, gas, CH4, microbial protein proxies	Links chemistry to rumen function
Microbiome	16S bacteria/archaea, protozoa or fungi when possible, alpha/beta diversity and taxa associations	Tests functional-feed mechanism
Animal outcomes	BW, ADG, BCS, milk or meat traits, oxidative biomarkers, welfare indicators	Determines practical value and safety
Parasite outcomes	Fecal egg counts, larval development, packed cell volume, clinical signs	Tests anti-H. contortus relevance
Sustainability and cost	Harvest cost, fuel, processing energy, replacement value, control effect, no-spread compliance	Determines feasibility and circularity

**Table 13:** Recommended design standards for controlled *R. ulmifolius* sheep trials.

Design Component	Weak Approach	Publishable Approach
Feed description	Plant identified only by species name	Voucher specimen, site, season, phenological stage, fraction, processing and batch chemistry
Control diet	Basal diet only	Basal control plus nutrient-matched and/or tannin-mechanism control when feasible
Dose selection	One arbitrary inclusion level	At least two levels with rationale based on tannin and fiber exposure
Adaptation	No adaptation or unspecified period	Defined adaptation with daily intake and refusal monitoring
Endpoints	Body weight only	DMI, digestibility, rumen fermentation, microbiome, nitrogen, methane, health and performance
Statistics	Simple comparison without batch information	Power-aware design with repeated measures and batch-level characterization

**Table 14:** Testable translational hypotheses, required experiments and decision endpoints.

Hypothesis	Experimental Test	Critical Endpoint	Possible Interpretation
Early regrowth has better feed value than mature thicket biomass	Compare fractions and seasons across sites	CP:ADL ratio; IVDMD; tannin activity	Defines optimal harvest window
Moderate inclusion improves nitrogen partitioning	Protein-matched sheep trial	Ruminal NH3-N, BUN, urinary/fecal N	Supports functional protein-protection effect
Phenolics modulate rumen microbiome	Dose–response trial with sequencing	Bacterial/archaeal shifts plus VFA and digestibility	Mechanistic evidence only if linked to function
Methane decreases without performance penalty	In vivo methane trial	CH4/kg DMI and CH4/kg gain or milk	Valid mitigation only if productivity maintained
Parasite resilience improves	Parasite-challenge grazing or pen trial	FEC, PCV, ADG, clinical signs	Supports integrated parasite-management role
Harvest valorization supports control	Field plots with repeated harvest	Regrowth, biomass yield, native cover, feed quality	Connects invasive management with feed supply

## Data Availability

No new data were created or analyzed in this study. Data sharing is not applicable to this article.
